# Fibromuscular Dysplasia with Spontaneous Coronary Artery Disease Presenting as Acute Myocardial Infarction

**DOI:** 10.7759/cureus.1268

**Published:** 2017-05-23

**Authors:** Ali Farooq, Waseem Amjad, Ata Ur Rahim Bajwa, Hassaan Yasin, Rizwan Ali, Muhammad Pervaiz

**Affiliations:** 1 Internal Medicine, West Virginia University - Charleston Division; 2 Forest Hills Hospital, Northshore-Long Island Jewish Health System; 3 Cardiology, University of Missouri Kansas City (UMKC); 4 Cardiology, West Virginia University - Charleston Division

**Keywords:** fibromuscular dysplasia, spontaneous coronary artery dissection

## Abstract

A 40-year-old female presented to a rural hospital with crushing substernal chest pain. An initial electrocardiogram showed ST elevation in lead II and aVF with elevated troponin I. She was immediately transferred to a tertiary care hospital. An emergent coronary angiogram did not show any significant coronary artery disease. On the second day, the patient experienced recurrence of severe chest pain with ST elevations in leads I, aVL, V5-V6, ST depressions in V1-V3, T-wave inversion over V2-V5. The troponin I level increased to > 40 ng/ml (normal 0.0 to 0.04 ng/ml). An emergent angiogram was performed revealing local dissection of the mid to distal left main coronary artery and a totally occluded diagonal artery. It was deemed unsafe to perform percutaneous coronary intervention because it was a non-flow limiting left main coronary artery dissection and was difficult to cannulate with the guide catheter. Subsequently, an elective angiogram was performed after a 48-hour interval to evaluate the progression of dissection and to make a definitive decision for revascularization versus medical management. On the third angiogram, stenosis seen in the diagonal branch on the previous angiogram progressed to dissection, and local dissection of the left main coronary artery seen on the previous angiogram spontaneously resolved. The patient was symptom-free and hemodynamically stable. It was decided to manage the patient conservatively due to the spontaneous resolution of occlusion in the diagonal artery and dissection of the left main coronary artery. The patient was started on conservative medical treatment. A magnetic resonance angiography of the right internal carotid artery revealed a “string of beads” appearance, which confirmed the diagnosis of fibromuscular dysplasia. She was followed closely in the clinic and has remained asymptomatic for the past one year.

## Introduction

Spontaneous coronary artery dissection (SCAD) is an uncommon but serious complication of fibromuscular dysplasia (FMD). Coronary artery involvement in FMD arises less frequently as compared to other arteries. Acute myocardial infarction (AMI) as a presentation of FMD is believed to be rare. The infrequency with which AMI presents as an initial manifestation of FMD makes prompt diagnosis and treatment of this condition a formidable diagnostic challenge. Once SCAD is diagnosed on the angiogram, patients with hemodynamic stability and adequate coronary artery blood flow can be managed successfully with conservative treatment.

## Case presentation

**Figure 1:**
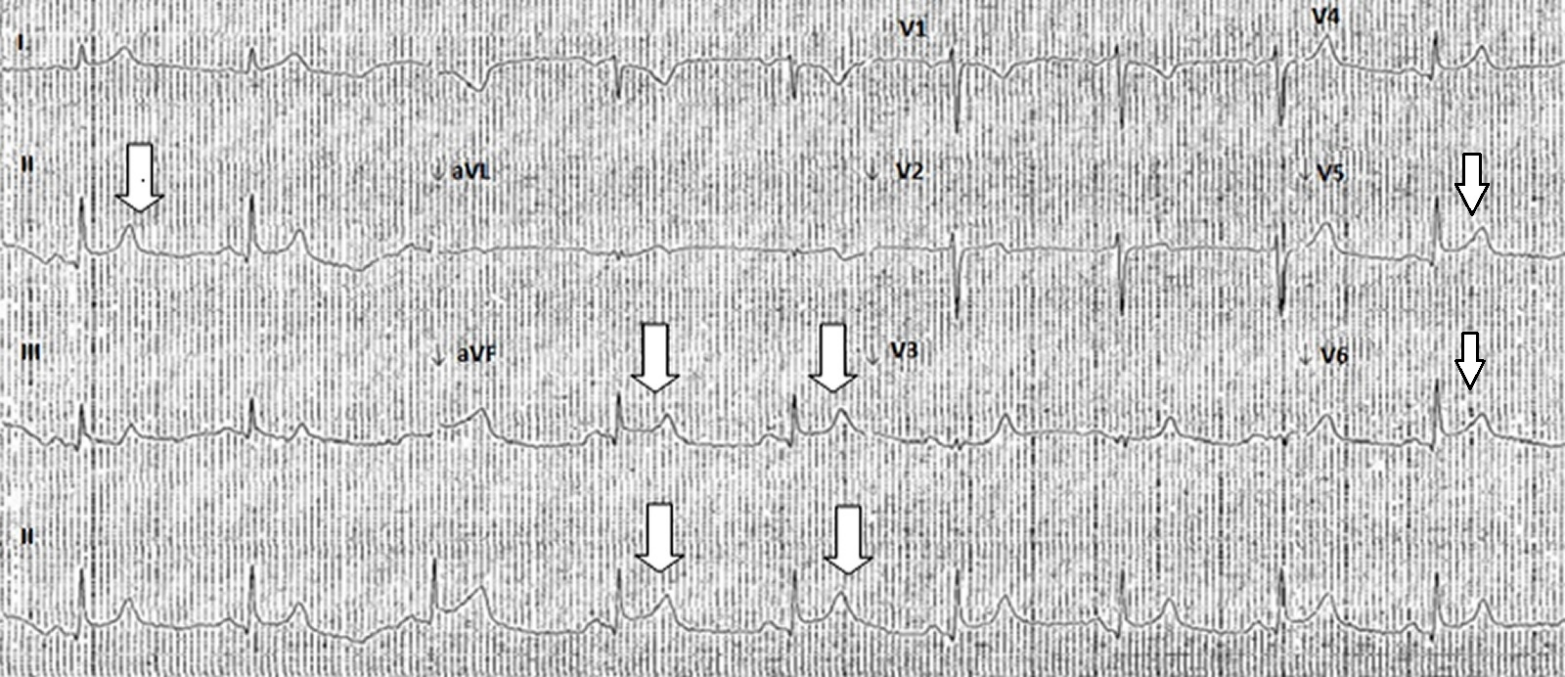
ST elevations in lead II, aVF, V5, and V6.

A 40-year-old female with no prior cardiac history presented to a rural hospital with crushing substernal chest pain for three-hour duration. The chest pain was aggravated with exertion and relieved with rest. She did not have any cardiovascular risk factors. Her social history was negative for smoking, alcoholism, or illicit drug use. There was no family history of premature coronary artery disease. Her only home medication was a multivitamin. She was hemodynamically stable, and there was no other abnormal finding on the physical exam. Her initial set of blood work at an outlying facility revealed a complete blood count and basic metabolic panel within normal limit, troponin I 11.3 ng/ml (normal 0.0 to 0.04 ng/ml). An electrocardiogram (EKG) obtained at an outlying facility showed ST elevations in lead II, aVF, V5 and V6 consistent with ST elevation myocardial infarction (STEMI) (Figure 1).

**Figure 2:**
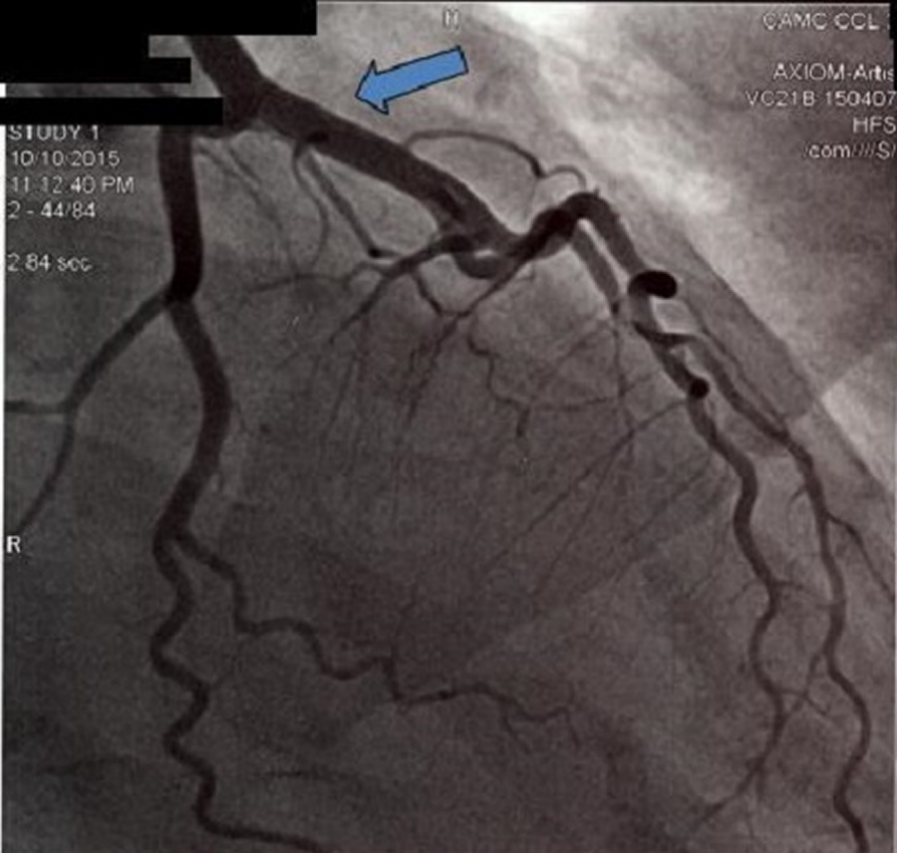
Emergent angiogram performed within first few hours of presentation showed patent left main coronary artery.

**Figure 3:**
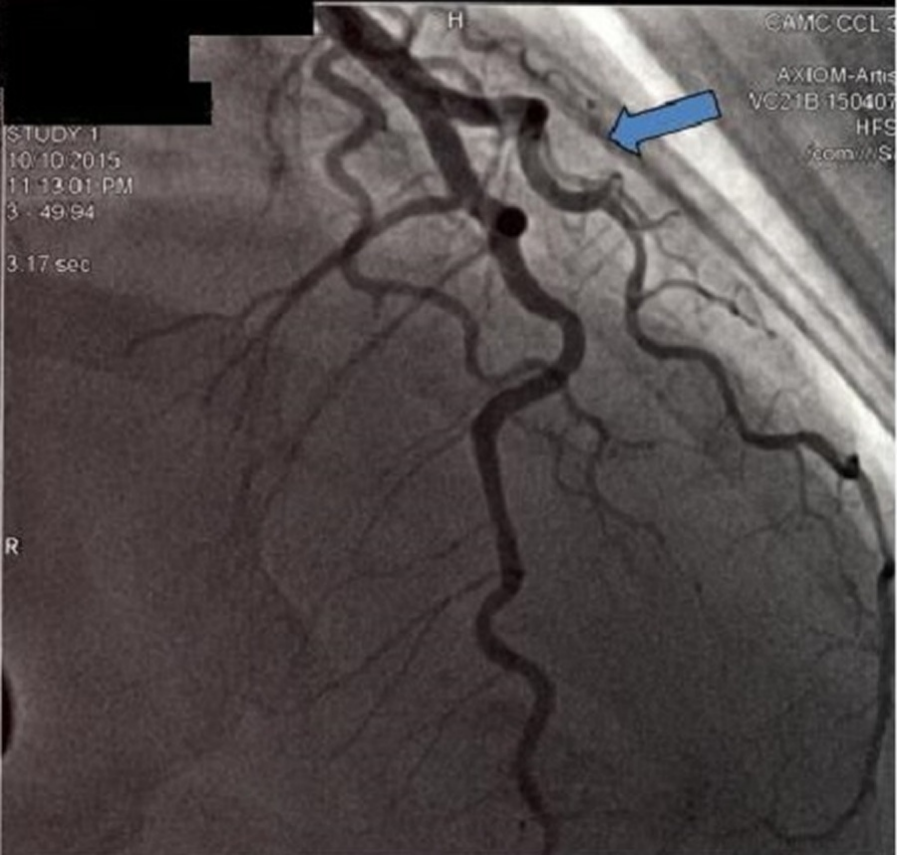
First angiogram revealed patent diagonal branch.

The patient was started on aspirin, beta blocker, statin and heparin drip. Because of the unavailability of percutaneous intervention (PCI) at the outlying facility, the patient was transferred to our facility. By the time of arrival at our facility, the patient was still experiencing chest pain. Emergent left heart catheterization was performed that showed normal left and right coronary arteries, patent left anterior descending (Figure 2) and patent diagonal artery (Figure 3).

**Figure 4:**
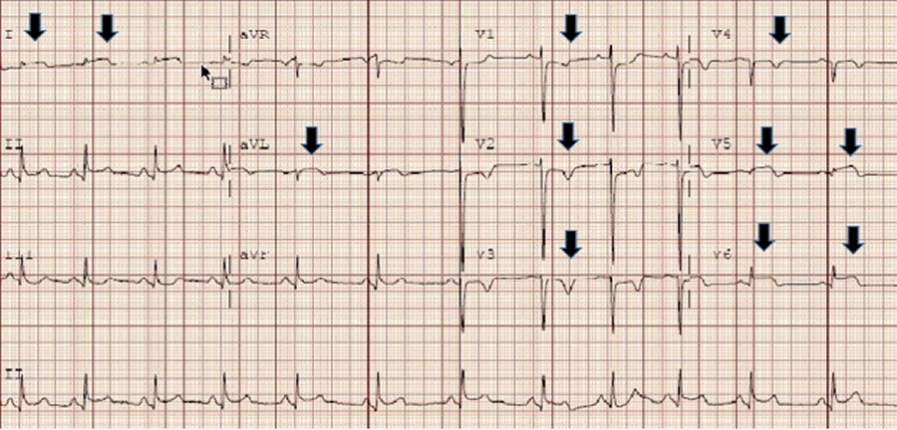
ST elevations in lead I, aVL, V5-V6, ST depressions in V1-V3, T-wave inversion over V2-V5.

The patient was transferred to the critical care unit for close monitoring. Her chest pain subsided and cardiac enzymes started trending down. After 24 hours of the initial angiogram, the patient experienced a recurrence of severe substernal chest pain. An EKG revealed ST elevations in lead I, aVL, V5-V6, ST depressions in V1-V3, and T-wave inversion over V2-V5 (Figure 4).

**Figure 5:**
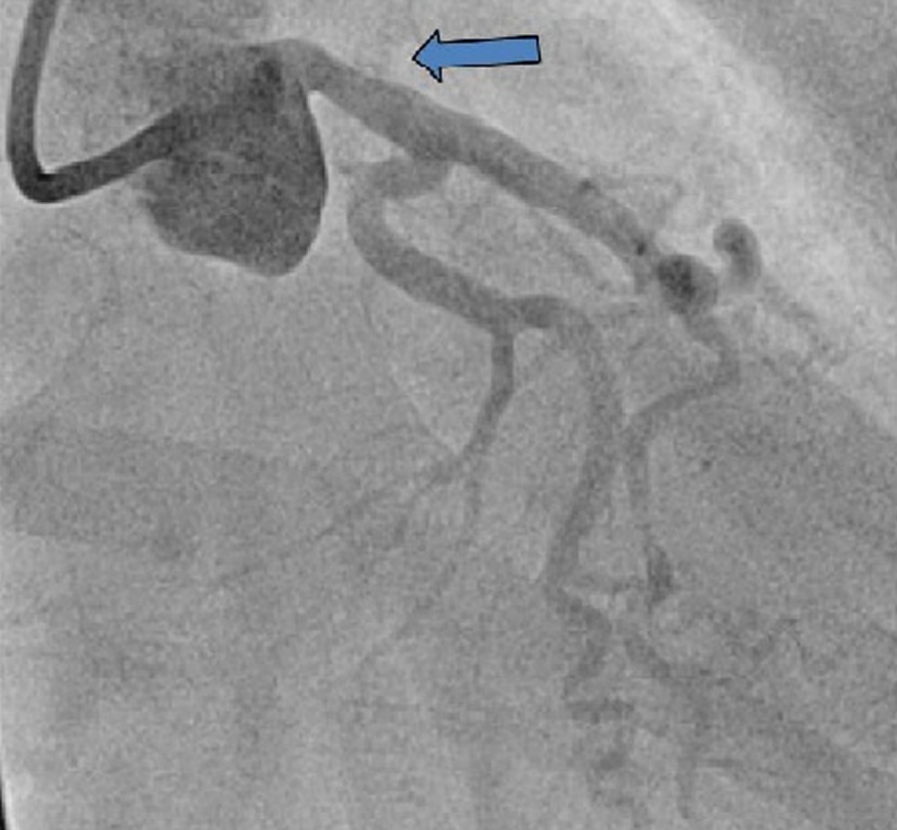
Second coronary angiogram revealed local dissection of mid to distal left main coronary artery that was not present on previous angiogram.

**Figure 6:**
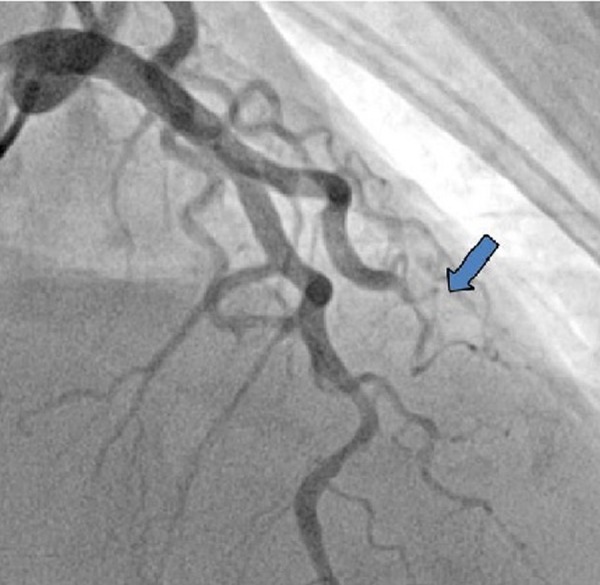
Second angiogram showed total occlusion of diagonal artery that was patent on previous angiogram.

The troponin I level increased to > 40 ng/ml (normal 0.0 to 0.04 ng/ml). She was emergently taken to the catheterization lab again. At this time, the coronary angiogram showed local dissection of the mid to distal left main coronary artery (LMCA) (Figure 5) and a total occlusion of the diagonal branch (Figure 6).

These changes were not present on the previous angiogram. It was deemed unsafe to proceed with any further intervention of local dissection of the LMCA because it was a non-flow limiting spontaneous LMCA dissection and difficult to cannulate with the guide catheter. It was planned to observe the patient for 48 hours and repeat angiography to evaluate the progression of dissection and to make a definitive decision for revascularization versus medical management.

**Figure 7:**
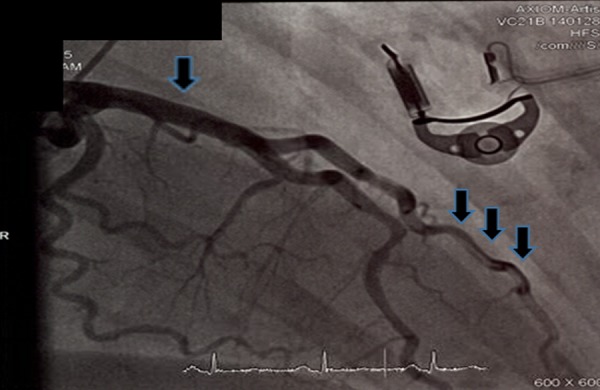
Third coronary angiogram showed spontaneous resolution of left main coronary artery dissection and long linear dissection of diagonal artery.

The patient was started on dual antiplatelet therapy with aspirin and clopidogrel and was continued on statin and beta-blocker therapy. Over the period of 48 hours, she was monitored closely in the critical care unit; her chest pain resolved and she remained hemodynamically stable. The EKG changes also resolved. The patient was brought back to the catheterization lab. Repeat angiography this time revealed that the local dissection of the LMCA seen on the previous angiogram spontaneously resolved. The total occlusion of diagonal artery seen on the previous angiogram was now replaced by a long linear dissection plane (Figure 7).

This suggests a SCAD, which was the likely culprit etiology for the patient’s initial occlusion of the diagonal branch on the previous angiogram. FMD was suspected as an underlying etiology of SCAD in this young female with no traditional risk factors for atherosclerotic disease. Further workup was done to confirm the diagnosis of FMD, and magnetic resonance angiography of the carotid arteries revealed a “string of beads” appearance in the right internal carotid artery, which confirmed the diagnosis of FMD.

At this point, the patient had spontaneous resolution of LMCA dissection and spontaneous conversion of total occlusion of the diagonal artery to non-flow limiting dissection. The patient was symptom-free and was hemodynamically stable; so a conservative approach was applied, and the patient was continued on optimal medical management with aspirin 81 mg, clopidogrel 75 mg, atorvastatin 40 mg, and metoprolol succinate 25 mg. The patient has been closely followed and over the course of one year has remained symptom-free.

## Discussion

Thirty percent of young females presenting with AMI have SCAD as an underlying etiology [1]. FMD has a very strong association with SCAD. Eighty-six percent cases of SCAD have concomitant FMD [2]. The pathophysiology of FMD causing SCAD is not completely known; however, it is suggested due to the genetic abnormality, fibrosis of the vasa vasorum leads to coronary vessel wall ischemia and proliferation of myofibroblasts resulting in dissection [3].

Clinicians should have a high suspicion for SCAD in young females without traditional risk factors for coronary artery disease presenting with AMI. Such patients should receive urgent angiography. However, angiography alone may not be sufficient to detect underlying SCAD if the involvement of coronary arteries is subtle. In such cases, new imaging modalities, intravascular ultrasound (IVUS) and optical coherent tomography (OCT) can be helpful to make the diagnosis of SCAD [4].

There are no clear guidelines for treatment of AMI secondary to SCAD. Current treatment strategies are based mostly on expert opinion and a few case series. Hemodynamically stable patients with adequate coronary artery blood flow and mild stenosis can be safely managed with a conservative approach. Medical management with antiplatelet therapy (aspirin, clopidogrel) and beta-blocker is appropriate. SCAD predisposes prothrombotic changes due to intimal tear of coronary arteries. Empiric dual antiplatelet therapy with aspirin and clopidogrel will be of potential benefit by preventing prothrombotic changes [5]. Aspirin and clopidogrel should be initiated immediately as soon as the diagnosis of SCAD is confirmed with angiography. Aspirin can be continued lifelong due to low side effects and low bleeding risks, and clopidogrel can be continued for up to one year. Beta-blocker therapy can reduce shear forces on the arterial wall by reducing blood pressure and heart rate. This reduction of shear forces on intimal tear will prevent extension of dissection. The role of statins in the management of SCAD has not been studied and should be considered in patients with preexisting dyslipidemia.

Anticoagulation therapy with intravenous (IV) heparin or enoxaparin can be potentially harmful in SCAD due to the risk of extension of intramural hematoma and dissection [6]. There is a lack of evidence-based data supporting the safety of anticoagulation in SCAD; therefore, anticoagulation should not be continued once the diagnosis of SCAD is confirmed on imaging.

Thrombolytic therapy is also generally avoided because of the risk of extension of dissection and intramural hematoma. In a retrospective review, 60% of SCAD patients who received thrombolytic agents deteriorated, requiring rescue PCI or coronary artery bypass graft (CABG) [7].

PCI in hemodynamically stable patients with adequate coronary artery blood flow should be avoided due to the potential risk of extension of the dissection. However, PCI should not be withheld in hemodynamically unstable patients or if dissection leads to severe vascular stenosis. With the success of bioresorbable vascular scaffolds (BVS) in the treatment of nonatherosclerotic CAD, BVS seems to be a better option in the treatment of SCAD presenting with STEMI or hemodynamic instability. BVS provides the transient scaffolding of the vessel wall, thus restoring arterial blood flow in vascular stenosis. Over time the BVS spontaneously resorbs into the vessel wall, thus reducing the potential risk of progression of the dissection as compared with conventional stenting. There is a reported case of SCAD involving the left circumflex artery successfully treated with BVS [8]. Due to the potential advantages of BVS over conventional stenting, there seems to be a promising role of BVS in the treatment of SCAD. However, there is a need for more studies to evaluate the benefit of BVS. CABG is indicated as a treatment option in patients with SCAD involving multiple vessels or patients with failed PCI.

## Conclusions

In hemodynamically stable patients with preserved coronary artery blood flow, conservative medical management should be opted first. However, revascularization with PCI, BVS, or CABG may be necessary for hemodynamically unstable patients or if dissection leads to severe vascular stenosis causing compromised myocardial blood flow.

It is crucial to identify SCAD from coronary atherosclerotic disease (CAD) as the etiology of AMI at an early stage because treatment of both conditions differs markedly. If initial angiography does not reveal SCAD, new imaging modalities such as IVUS and OCT can be helpful to identify subtle changes of SCAD from CAD and avoid unnecessary and potentially harmful management of CAD, for example, thrombolytic therapy and PCI.
